# A novel colorimetric sensing platform for the detection of *S. aureus* with high sensitivity and specificity†

**DOI:** 10.1039/c9ra05304b

**Published:** 2019-10-18

**Authors:** Yun Zhang, Shuyou Shi, Jiajia Xing, Wenqing Tan, Chenguang Zhang, Lin Zhang, Huan Yuan, Miaomiao Zhang, Jinjuan Qiao

**Affiliations:** Henan Key Laboratory of Immunology and Targeted Therapy, Henan Collaborative Innovation Center of Molecular Diagnosis and Laboratory Medicine, School of Laboratory Medicine, Xinxiang Medical University Xinxiang 453003 PR China zhangyun0126@126.com +86 373 3029977 +86 373 3029977; School of International Education, Xinxiang Medical University Xinxiang 453003 PR China; School of Innovation and Entrepreneurship, Xinxiang Medical University Xinxiang 453003 PR China; Department of Medical Laboratory, Weifang Medical University Weifang 261053 PR China

## Abstract

In this study, a novel colorimetric sensing platform was developed for the detection of *S. aureus* using dog immunoglobulin G (IgG) as the capture antibody and chicken anti-protein A immunoglobulin Y labeled with horseradish peroxidase (HRP-IgY) as the detection antibody. Dog IgG labeled with magnetic beads was used to capture *S. aureus* through the interaction between the Fc region of dog IgG and Staphylococcal protein A (SPA). HRP-IgY was introduced to recognize the residual SPA on the surface of *S. aureus* and to create a sandwich format, after which a soluble 3,3′,5,5′-tetramethylbenzidine (TMB) substrate was added. A stop solution was utilized to cease the enzymatic chromogenic reaction, and then optical density was read at 450 nm. Under optimal conditions, the proposed method displayed a low detection limit of 1.0 × 10^3^ CFU mL^−1^ and a wide linear range of 3.1 × 10^3^ to 2.0 × 10^5^ CFU mL^−1^. This detection method exhibited high specificity against other foodborne bacteria. The recovery rates ranged from 95.2% to 129.2%. To our knowledge, this is the first report to employ dog IgG and chicken IgY as an antibody pair to detect *S. aureus*. This technique exhibits high application potential for *S. aureus* monitoring in various kinds of samples.

## Abstract 2

Utilization of dog IgG and chicken anti-protein A IgY as an antibody pair for sensitive and selective detection of *S. aureus*.

## Graphical abstract

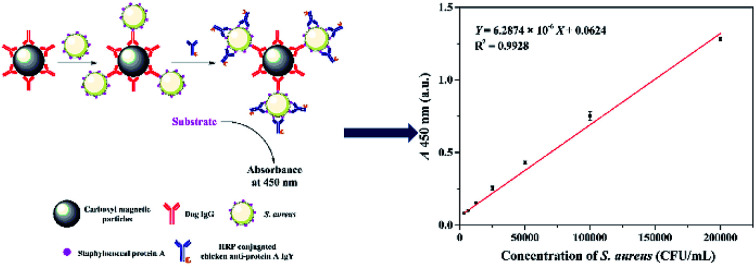

## Introduction

*Staphylococcus aureus* (*S. aureus*) is one of the most common food-borne pathogens, and can cause various infections ranging from scalded skin syndrome to life-threatening septicemia.^1–3^ Annually, *S. aureus* causes about 241 000 cases of food-borne illnesses, which pose a significant threat to public health and food safety.^4,5^ It is therefore important to develop effective detection strategies for *S. aureus*.

To date, several detection strategies have been developed to monitor *S. aureus*. Among them, the conventional culture-based method is considered as the gold standard. This method relies on multiple steps to obtain a sufficient number of bacterial cells prior to biochemical assays.^6,7^ Although the culture-based method is simple, the total detection procedure time required is longer than 2 days, making it unsuitable for rapid diagnosis of *S. aureus*. Polymerase chain reaction (PCR)-based approaches have been utilized to detect specific genes of *S. aureus* with high sensitivity and specificity.^8,9^ However, they are limited by the complex matrix, long-term bacterial enrichment, low bacterial abundance and sample cross-contamination.^10^ Enzyme linked immunosorbent assay (ELISA) can couple the amplification effect of enzymatic reactions with selective antigen–antibody binding and increase the sensitivity and specificity of the assay. Due to its low-cost, high sensitivity and high throughput application, ELISA is the most frequently used method for antigen detection.^11,12^ Nevertheless, few ELISA-based methods have been used to detect *S. aureus* cells. This is likely due to the lack of suitable antibody pairs, which limit the development of specific immunoassays for *S. aureus*.^13^ Since mammalian monoclonal or polyclonal antibodies against *S. aureus* can also bind to protein G producing *Streptococcus* by interactions between the Fc region of mammalian Immunoglobulin G (IgG) and protein G,^14^ the specificity of detection is not guaranteed. Non-specific interactions of the bio-recognition element with target analytes lead to inaccurate results in the detection process.^15^ Thus, it is of particular significance to select a feasible antibody pair for an ELISA-based detection of *S. aureus*.

In the present study, a novel colorimetric sensing platform was developed using dog IgG and chicken IgY as an antibody pair to detect *S. aureus*. Primarily, Staphylococcal protein A (SPA) was used as an ideal detection marker of *S. aureus*, since SPA is only expressed in the cell wall of 99% *S. aureus* isolates and the number of SPA molecules is estimated to approximately 80 000 in each bacterium.^16–19^ Dog IgG was utilized as the capture antibody that binds to *S. aureus* based on the strong affinity between the Fc region of dog IgG and SPA. Since dog IgG possesses strong binding capacity towards protein A and low binding capacity towards protein G,^20,21^ it can eliminate the interference from protein G producing *Streptococcus* to a certain extent. Moreover, chicken anti-protein A IgY was adopted as the detection antibody to further increase the specificity, because chicken anti-protein A IgY does not exhibit non-specific binding to protein G.^22–25^ Therefore, the proposed antibody pair could be used to improve the selectivity of ELISA.

**Fig. 1:**
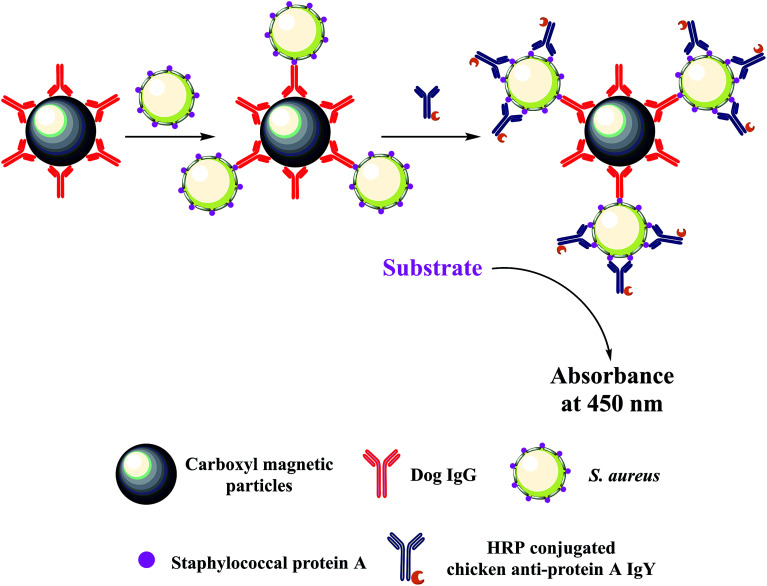
Schematic illustration of the novel colorimetric sensing platform for the detection of *S. aureus* (not to scale).

Immunomagnetic beads based ELISA (IMBs-ELISA) is developed by combining ELISA with magnetic separation.^26–28^ IMBs can be easily separated from the reaction mixture with a magnet and re-dispersed immediately following removal of the magnet, which allow for a nearly “in solution” reaction.^12,29^ These characteristics render IMBs-ELISA more efficient and sensitive. Therefore, the aim of the present study was to establish an IMBs-ELISA method that could combine with the high sensitivity of IMBs-ELISA and the high specificity of the novel antibody pair. Dog IgG coated on magnetic beads (MBs) and horseradish peroxidase (HRP) conjugated chicken anti-protein A IgY were utilized to bind with SPA on the surface of *S. aureus* in a sandwich structure (as illustrated in Fig. 1). Following enzymatic amplification, the optical density was measured and a standard curve was generated for quantitative analysis of *S. aureus*. Moreover, the proposed method was applied to detect the number of bacteria in spiked samples and validate the potential application of this method in real samples.

## Experimental

### Reagents and materials

*S. aureus* (ATCC 12598), *S. aureus* (ATCC 25923), *S. aureus* (ATCC 139843), *E. coli* O157:H7 (EDL 933), *Salmonella* (ATCC 14028) and *Listeria monocytogenes* (ATCC 19115) were obtained from BeNa Culture Collection (Beijing, China). *Streptococcus agalactiae* (isolated from raw milk) was gifted from Prof. Hongping Wei, Wuhan Institute of Virology, Chinese Academy of Science. Carboxylated MBs with a 0.82 μm diameter (25 mg mL^−1^) were purchased from Spherotech (USA). *N*-(3-Dimethylaminopropyl)-*N*′-ethylcarbodiimide hydrochloride (EDC) and *N*-hydroxysuccinimide (NHS) were bought from Aladdin Industrial Corporation (Shanghai, China). Dry powder of dog IgG (10 mg, dissolved in 10 mL of phosphate-buffered saline (PBS) (10 mmol L^−1^, pH 7.4)), streptavidin-Cy5.5 (1 mg mL^−1^) and Tween-20 were supplied by Solarbio (Beijing, China). HRP conjugated chicken anti-protein A IgY (HRP-IgY) (1 mg mL^−1^) was purchased from Abcam (England). Soluble 3,3′,5,5′-tetramethylbenzidine (TMB) substrate was procured from Biodragon Immunotechnologies (Beijing, China). Bovine serum albumin (BSA) and skim milk powder were purchased from Biosharp (Hefei, China) and BD Difco (USA), respectively. 96 well flat-bottomed microplates were obtained by Jet bio-filtration (Guangzhou, China). The aqueous solutions were prepared in ultrapure water (18.2 MΩ) obtained from Ulupure UPR-II-10T (Sichuan, China). All other chemicals and reagents purchased from commercial sources were of the highest purity grade. Orange juice and spring water were procured from a local supermarket, and human urine samples were collected from healthy volunteers. All experiments were performed in compliance with the relevant laws of China and institutional guidelines of Xinxiang medical university. Volunteers signed an informed consent approved by the ethics committee of Xinxiang medical university.

### Preparation and culture bacteria

*S. aureus*, *E. coli* O157:H7 and *Salmonella* were prepared by growing the stock cultures in Luria–Bertani broth medium, and *Streptococcus agalactiae* and *Listeria monocytogenes* were cultured in the brain heart infusion broth medium. Following incubation at 37 °C for 12 to 16 h, the bacteria were collected by centrifugation at 3000 rpm for 10 min and subsequently washed twice by sterile PBS (10 mmol L^−1^, pH 7.4). The bacterial precipitate was re-suspended in sterilized PBS (10 mmol L^−1^, pH 7.4) containing 20% glycerol and the bacterial solutions were stored at −20 °C until further use. The concentration of bacteria was determined by conventional colony counting method. The bacterial solutions were diluted 10-fold with sterile PBS and 100 μL of these samples were incubated with the corresponding medium agar at 37 °C overnight. The number of colonies formed on the plates was counted to calculate the bacterial concentration.

### Preparation of IMBs

Bio-conjugation of carboxyl groups of the MBs with the amino-groups of the dog IgG was achieved *via* a classic EDC/NHS amidization reaction.^30^ Briefly, 1 mg of MBs was separated using magnetic separation racks (Goldmag Biotech, China) to remove the storage buffer. The MBs were subsequently washed twice with PBS (pH 6.8) by a magnetic separation process. A total of 2 mg of EDC and 1 mg of NHS were dissolved in 200 μL of PBS (10 mmol L^−1^, pH 6.8) and were utilized to activate the carboxyl groups of the MBs. Following 30 min of activation, 100 μL of dog IgG (1 mg mL^−1^) was added to the MBs. The samples were incubated on a rotating platform (180 rpm) at 37 °C for 4 h to form MBs-dog IgG conjugates. Afterwards, 1 mL of 5% BSA and 15% skim milk was added to block non-specific sites on MBs for an additional 2 h and the samples were washed twice. Finally, the IMBs were re-suspended in 1 mL of PBS containing 1% BSA and stored at 4 °C.

### Detection procedure

A typical sandwich assay was performed in Eppendorf (EP) tubes fitted to the magnetic separation racks. Each incubation step was processed under shaking conditions (180 rpm) at 37 °C. 20 μL of IMBs (1 mg mL^−1^) was pipetted into each tube and then 100 μL of *S. aureus* solution was added. The mixture was incubated for 30 min and the beads were subsequently secured by placing the EP tube on the magnetic separation racks. Following removal of the supernatant, the beads were rinsed five times with PBST (PBS with 0.05% Tween-20). Subsequently, the MBs-dog IgG-*S. aureus* complex was dispersed in 100 μL of HRP-IgY (0.5 μg mL^−1^) and the entire mixture was rotated for 30 min. HRP-IgY formed a sandwich structure with *S. aureus* and MBs-dog IgG by an immune reaction. The immune sandwich complex was separated magnetically and the supernatant was discarded. The immune complex was rinsed five times with PBST to effectively remove unbound HRP-IgY. Thereafter, 100 μL of soluble TMB substrate was added into each EP tube, followed by incubation for 10 min. The reaction was terminated by the addition of 100 μL of stop solution (2 mol L^−1^ sulfuric acid). Following magnetic separation, 100 μL of the supernatant was transferred to the 96 well plate and the absorbance at 450 nm was determined by the Varioskan Flash multimode reader (Thermo Scientific, USA).

## Results and discussion

### Optimization of experimental components

**Fig. 2:**
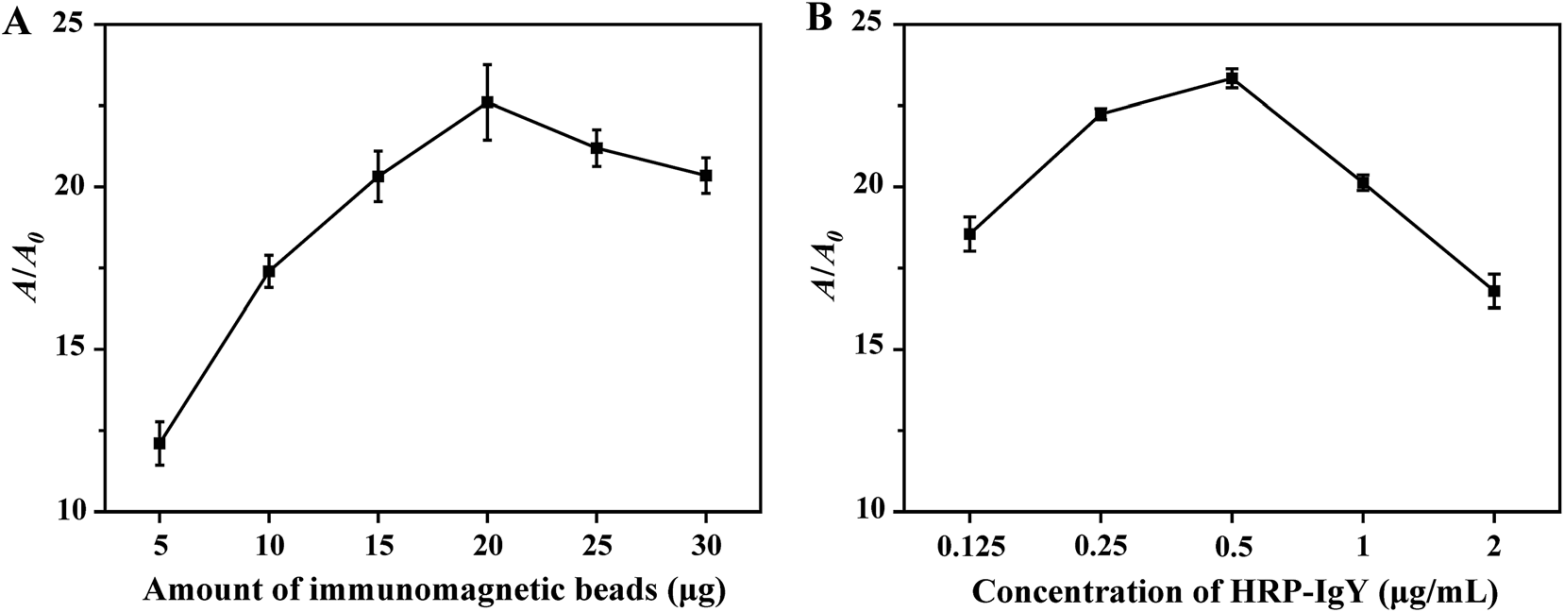
Effects of experimental components on *A*/*A*_0_ of the system. (A) Effects of the amount of IMBs addition on *A*/*A*_0_. (B) Effects of the concentration of HRP-IgY on *A*/*A*_0_. Three independent measurements were taken from three individual preparations for each condition. Error bars indicated the standard deviations.

Initially, fluorescence microscopy was used to characterize the binding of dog IgG towards *S. aureus*. The latter was stained with fluorescence using streptavidin-Cy5.5 linked with biotin-dog IgG. The biotin–streptavidin reaction was used in order to demonstrate whether dog IgG could bind to *S. aureus* cells. The blue colour of the precipitate was noted at the bottom of the EP tubes following centrifugation, indicating the attachment of dog IgG to *S. aureus* (as shown in ESI Fig. S1A†). In addition, the stained bacteria were observed by fluorescence microscope (Pannoramic DESK P-MIDI, 3DHISTECH), and the fluorescence images illustrated strong red fluorescent emission from Cy5.5 on the cells of *S. aureus*, demonstrating the binding of dog IgG to *S. aureus* (as illustrated in ESI Fig. S1B†). Subsequently, the experimental components were optimized. In IMBs-ELISA, the dose of IMBs was a key determinant of the detection sensitivity of the proposed assay. Therefore, it was important to optimize the amount of IMBs added to the IMBs-ELISA. In Fig. 2, the parameters *A* and *A*_0_ represented the absorbance intensity at 450 nm for 5.0 × 10^4^ CFU mL^−1^*S. aureus* and for a blank of PBS, respectively. As shown in Fig. 2A, *A* and *A*_0_ were gradually increased with increasing amounts of IMBs. This may be attributed to non-specific binding of HRP-IgY onto IMBs caused by the increase in the amount of IMBs. Therefore, in order to obtain an optimal balance between high detection sensitivity and low nonspecific signal, optimal *A*/*A*_0_ was achieved at 20 μg of IMBs addition (as illustrated in ESI Fig. S2A†). 20 μg of IMBs was selected for the subsequent tests.

The process of the colorimetric assay is mainly derived from HRP-catalyzed oxidation of TMB. For this reason, the concentration of HRP-IgY in the proposed method should be optimized. As illustrated in Fig. 2B, *A* and *A*_0_ were increased at higher HRP-IgY concentration. The increase in *A*_0_ was faster than that of *A* when the concentration of HRP-IgY was higher than 0.5 μg mL^−1^. This result suggested that an HRP-IgY concentration higher than 0.5 μg mL^−1^ exceeded the amount required for target capture, which led to nonspecific absorption of HRP-IgY onto IMBs, thereby increasing the background signal. As presented in ESI Fig. S2B,† a strong signal intensity with relatively lower background signal was recorded when the HRP-IgY concentration was 0.5 μg mL^−1^. Therefore, 0.5 μg mL^−1^ was determined to be the optimal concentration of HRP-IgY.

### Detection of *S. aureus* in PBS

**Fig. 3:**
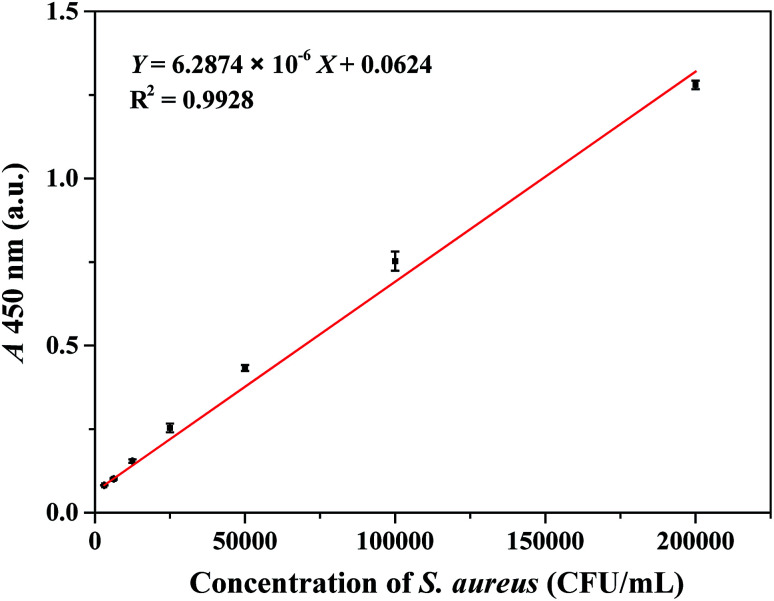
Calibration curve of absorbance intensity (450 nm) along with *S. aureus* concentration under the optimal conditions in PBS. Three independent measurements were taken from three individual preparations for each condition. Error bars indicated the standard deviations.

Under optimal conditions, a serial concentration of *S. aureus* was tested ranging from 3.1 × 10^3^ to 2.0 × 10^5^ CFU mL^−1^. As shown in Fig. 3, a calibration curve was developed based on the relationship between the different concentrations of *S. aureus* and the absorbance intensity at 450 nm. The linear regression equation was the following: *Y* = 6.2874 × 10^−6^*X* + 0.0624 (*R*^2^ = 0.9928), where *Y* and *X* represented the absorbance intensity (450 nm) of the reaction solution and the concentration of *S. aureus* (CFU mL^−1^), respectively. The detection limit (3*σ*, where *σ* is the standard deviation of PBS processed similarly as the *S. aureus* samples, *n* = 10, data were provided in ESI Table S1†) was calculated to be 1.0 × 10^3^ CFU mL^−1^. The total detection procedure was completed in less than 90 min. The reproducibility of the colorimetric immunoassay was tested using 2.0 × 10^5^ CFU mL^−1^*S. aureus*. The variability data of 11 replicates were obtained. As listed in ESI Table S2,† the relative standard deviation (RSD) was 4.12%, which demonstrated that the assay exhibited optimal reproducibility and reliability for *S. aureus* detection.

In order to test the robustness of the proposed method, the IMBs capturing *S. aureus* were performed in different pH values and PBS concentrations corresponding to the buffer solutions. As shown in ESI Fig. S3 and S4,† the IMBs exhibited ideal performance in the buffer solution over a wide range of the pH values (pH 4–9), and the PBS concentrations of the buffer solutions did not significantly affect the experimental results.

The satisfactory detection sensitivity was attributed to the following factors. MBs functioning as the immobile phase exhibited a large surface area, which resulted in the immobilization of a high number of antibodies and the efficient capture of *S. aureus*. In addition, *S. aureus* was captured by MBs through the interaction between SPA and dog IgG during the primary incubating step. A limited number of SPA molecules were occupied by antibodies due to steric hindrance between magnetic particles (∼0.82 μm) and *S. aureus* (∼0.8 μm). Given that one cell of *S. aureus* contains several SPA molecules and that the HRP-IgY antibodies exhibit relatively small molecular size, many residual SPA molecules could be recognized by HRP-IgY, thereby amplifying the colorimetric signal. The proposed IMBs-ELISA indicated comparable sensitivity with previous colorimetric methods for *S. aureus* detection.^13,31–35^ It possessed the advantages of not utilizing rare metals, complicated nanomaterial preparations, trained staff or sophisticated instruments, and all reagents were cheap and commercially available.

### Detection of *S. aureus* in spiked samples

The effectiveness of the proposed detection system was evaluated on spiked samples. Briefly, stock solutions of *S. aureus* were diluted with orange juice, spring water and human urine, respectively. Standard curves were generated (as shown in ESI Fig. S5–S7†). The detection limits for orange juice, spring water and human urine were all estimated to 1.5 × 10^3^ CFU mL^−1^, indicating that the sensitivity of this assay was sufficient to detect low concentration of bacteria in real samples.^13,36,37^ The results of the spike-recovery study were provided in Table 1. As seen from this table, the recovery percentage of *S. aureus* tested by the assay ranged from 95.2% to 129.2%. As listed in ESI Table S3,† the results obtained from the present method were consistent with those obtained from the ELISA method, which further demonstrated the suitability of the quantitative assay for testing various types of samples. These results demonstrated that the proposed method was robust and was effective in detecting *S. aureus* in real samples.

**Table 1:** Recoveries of *S. aureus* spiked in real samples (*n* = 3)

Samples	Added amount (CFU mL^−1^)	Found amount (CFU mL^−1^)	RSD (%)	Recovery (%)
Orange juice 1^a^	6.3 × 10^3^	8.1 × 10^3^	5.0	129.2
Orange juice 2^a^	2.5 × 10^4^	3.0 × 10^4^	2.9	119.0
Orange juice 3^a^	1.0 × 10^5^	1.1 × 10^5^	7.3	106.4
Spring water 1	6.3 × 10^3^	6.3 × 10^3^	0.8	100.3
Spring water 2	2.5 × 10^4^	2.4 × 10^4^	1.9	95.2
Spring water 3	1.0 × 10^5^	1.1 × 10^5^	2.0	111.4
Human urine 1^b^	6.3 × 10^3^	6.5 × 10^3^	7.9	103.6
Human urine 2^b^	2.5 × 10^4^	2.6 × 10^4^	3.4	104.0
Human urine 3^b^	1.0 × 10^5^	1.0 × 10^5^	4.7	101.9

a The sample was 5 times diluted.

b The sample was 10 times diluted.

### Specificity

**Fig. 4:**
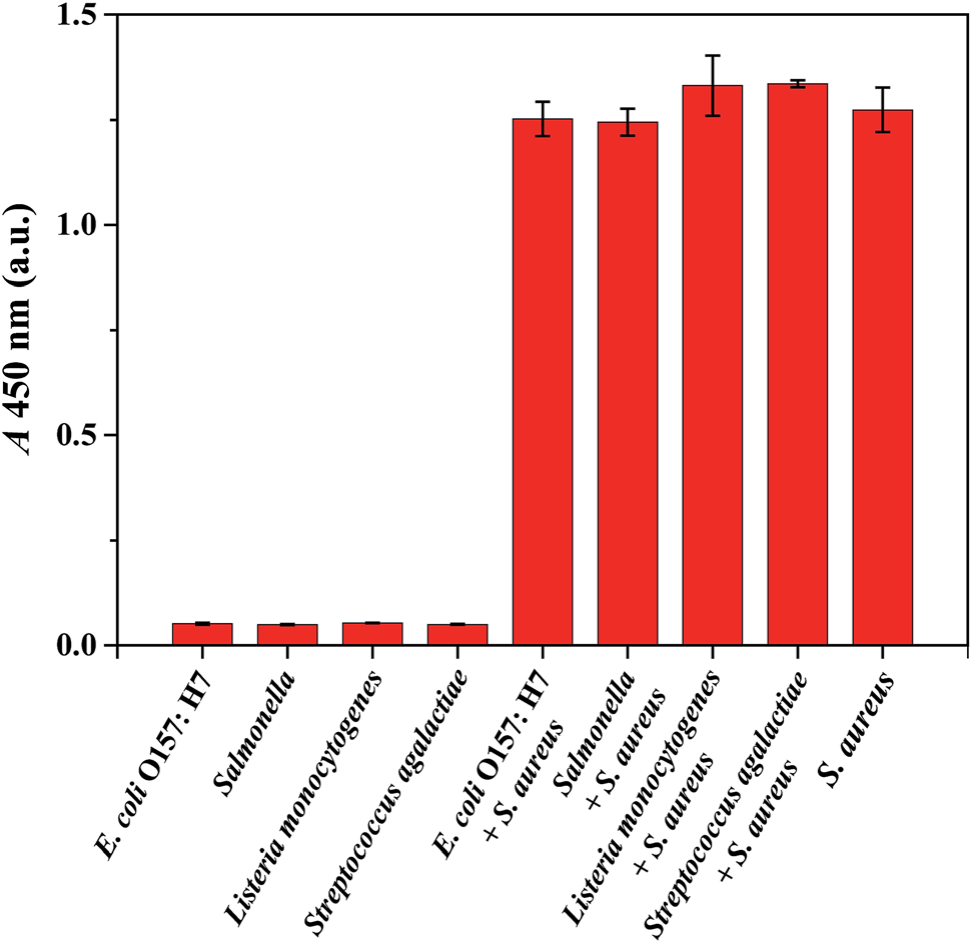
The specificity study for the proposed method for *S. aureus* detection. Red bars indicated absorbance intensity (450 nm) (from left to right) for 2.0 × 10^6^ CFU mL^−1^*E. coli* O157:H7, *Salmonella*, *Listeria monocytogenes* and *Streptococcus agalactiae*; *E. coli* O157:H7, *Salmonella*, *Listeria monocytogenes* and *Streptococcus agalactiae* (2.0 × 10^6^ CFU mL^−1^) interfered with 2.0 × 10^5^ CFU mL^−1^*S. aureus*; *S. aureus* (2.0 × 10^5^ CFU mL^−1^). Three independent tests were performed on three individual preparations for each condition. Error bars indicated the standard deviations.

The specificity of the proposed IMBs-ELISA in detecting *S. aureus* was evaluated against various non-target pathogenic bacteria (*E. coli* O157:H7, *Salmonella*, *Listeria monocytogenes* and *Streptococcus agalactiae*). As illustrated in Fig. 4, *S. aureus* at 2.0 × 10^5^ CFU mL^−1^ and its mixture with non-target pathogenic bacteria (all at 2.0 × 10^6^ CFU mL^−1^) displayed a high intensity signal, while non-target pathogenic bacteria produced negative results since they lack protein A in their cell wall. Negligible interference from *Streptococcus* was observed since dog IgG has weak binding affinity towards protein G, and chicken anti-protein A IgY reacts solely with protein A. These results suggested high specificity of the assay for *S. aureus* as a result of the highly specific recognition between the novel antibody pair and *S. aureus*.

SPA has been utilized as a detection marker for *S. aureus* in the agglutination test.^38^ Recently, several methods relying on the recognition of SPA have been put forwarded for the detection of *S. aureus*.^17,32,39,40^ For example, a chemiluminescent method for *S. aureus* detection was established by utilizing SPA-modified magnetic beads as competitive bacterial analogues and HRP labeled IgG as tracer, which could be completed in 50 min with a low detection limit of 6 CFU mL^−1^,^17^ while the presence of protein G producing *Streptococcus* would also result in positive detection signals. Dual-recognition strategies for *S. aureus* detection were based on vancomycin-affinity, utilizing IgG as the second recognition agent to bind with SPA.^32,39^ Vancomycin is a type of broad-spectrum antibiotics that can bind with multiple Gram-positive bacteria. Therefore these methods were expected to be prone to interference from Gram-positive bacteria. Chicken anti-protein A IgY was previously utilized as an antibody pair for the quantitative detection of *S. aureus* with high sensitivity and selectivity.^40^ Despite its desired selectivity, the consumption of chicken anti-protein A IgY that served as capture antibody was relatively high, resulting in higher cost of the test. To lower the test cost, dog IgG was introduced as a capture agent to detect *S. aureus* in the present study. However, there is a major limitation of these proposed SPA recognition-based methods is caused by the different expression levels of SPA genes noted among different strain types (as shown in ESI Fig. S8†). In addition, the different growth phases of *S. aureus* must be taken into consideration.^41^ In some cases, these SPA recognition based methods could be only used to detect *S. aureus* qualitatively.

## Conclusion

Owing to the large number of SPA molecules on one *S. aureus*, dog IgG and HRP-IgY were used as the capture antibody and detection antibody, respectively, to develop a novel colorimetric sensing platform for highly sensitive and specific detection of *S. aureus* in this study. A low detection limit of 100 CFU of *S. aureus* in 100 μL of PBS without enrichment, with a linear range from 3.1 × 10^3^ to 2.0 × 10^5^ CFU mL^−1^ was obtained. If the enrichment of magnetic beads is considered, the detection limit can be much lower (10 CFU in 100 μL can be determined if the detection volume is 1 mL). The whole detection procedure was completed within 90 min. Moreover, the method built here circumvented the interference of protein G generating *Streptococcus* due to the advantage of the novel antibody pair being highly selective and no cross-reactivity with other food-borne bacteria. The proposed IMBs-ELISA method presents an important platform that can be applied to develop a commercial kit for *S. aureus* screening.

## Conflicts of interest

There are no conflicts to declare.

## Supplementary Material

RA-009-C9RA05304B-s001
