# Supplementary material for: A novel colorimetric sensing platform for the detection of S. aureus with high sensitivity and specificity
Source: RSC Adv. 2019 Oct 18;9(58):33589–95. doi: 10.1039/c9ra05304b (PMC9073649; doi:10.1039/c9ra05304b)
Supplement: RA-009-C9RA05304B-s001 [file RA-009-C9RA05304B-s001.pdf]

Electronic Supplementary Material (ESI) for RSC Advances.
This journal is © The Royal Society of Chemistry 2019

Supplementary Information

A novel colorimetric sensing platform for the detection of *S. aureus* with high sensitivity and specificity

Yun Zhang [a, 1, *], Shuyou Shi [a, 1], Jiajia Xing [b], Wenqing Tan [a], Chenguang Zhang [a], Lin Zhang [c], Huan Yuan [a], Miaomiao Zhang [a], Jinjuan Qiao [d]

[a] Henan Key Laboratory of Immunology and Targeted Therapy, Henan Collaborative Innovation Center of Molecular Diagnosis and Laboratory Medicine, School of Laboratory Medicine, Xinxiang Medical University, Xinxiang 453003, PR China

[b] School of International Education, Xinxiang Medical University, Xinxiang 453003, PR China

[c] School of Innovation and Entrepreneurship, Xinxiang Medical University, Xinxiang 453003, PR China

[d] Department of Medical Laboratory, Weifang Medical University, Weifang 261053, PR China

[1] these authors contributed equally to this work

[*]Corresponding authors: Yun Zhang, Tel: +86 373 3029977; Fax: + 86 373 3029977; Email: zhangyun0126@126.com

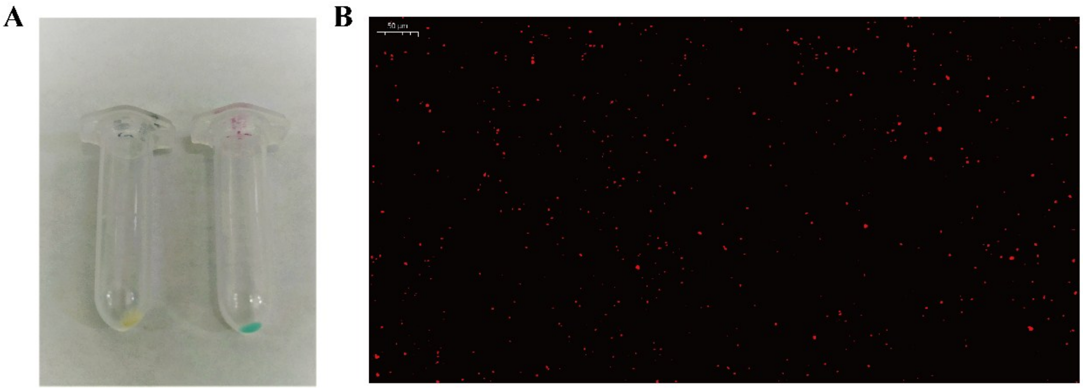

Fig. S1 (A) The image of blue bacteria pellet of Cy5.5 binding *S. aureus* after centrifuge. (B) Fluorescence micrographs of Cy5.5-stained *S. aureus*. Bar size: 50 μm

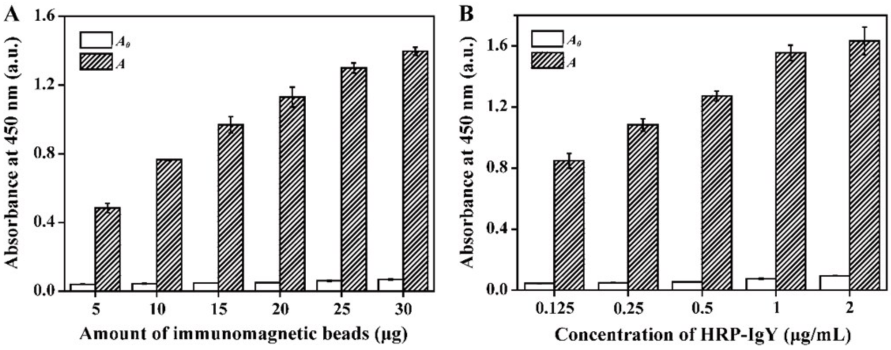

Fig. S2 Effects of experimental conditions on signals of the system. Three independent measurements were taken from three individual preparations for each condition. Error bars indicated the standard deviations

Table S1 Standard deviation test data of negative control

No.	Absorbance at 450 nm (a.u.)
1	0.049
2	0.05
3	0.051
4	0.051
5	0.052
6	0.052
7	0.052
8	0.052
9	0.052
10	0.057

Table S2 Relative standard deviation test data for reproducibility

No.	Absorbance at 450 nm (a.u.)
1	1.218
2	1.22
3	1.222
4	1.241
5	1.241
6	1.267
7	1.267
8	1.293
9	1.334
10	1.250
11	1.356

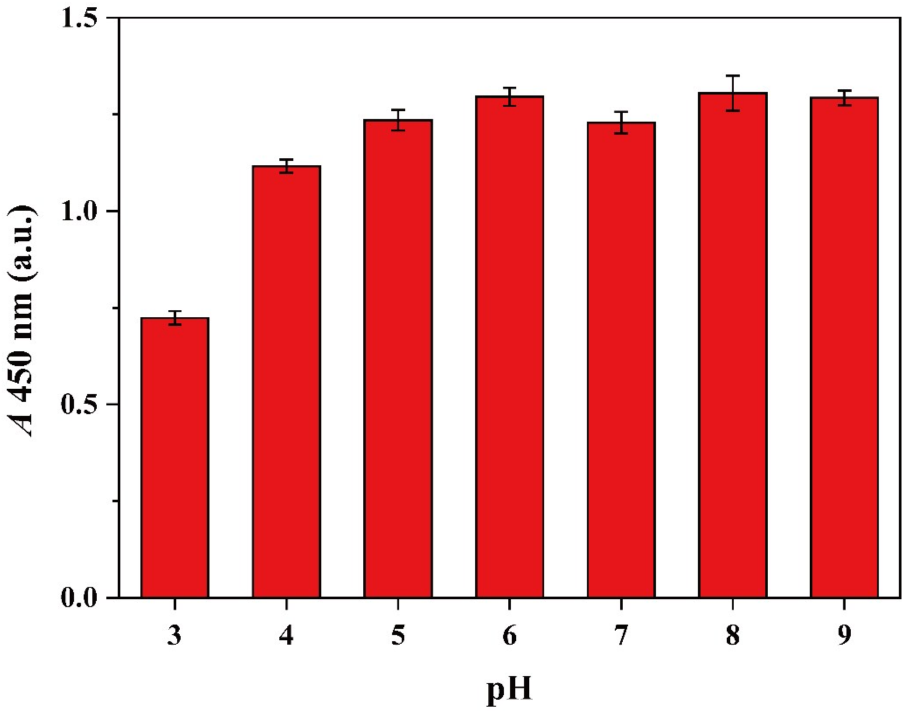

Fig. S3 Effect of pH on the performance of the proposed method. Red bars indicated absorbance intensity (450 nm) (from left to right) for 2.0 × 10^5 CFU/mL *S. aureus* in buffer solutions over a range of pH values 3-9. Error bars indicated the standard deviations

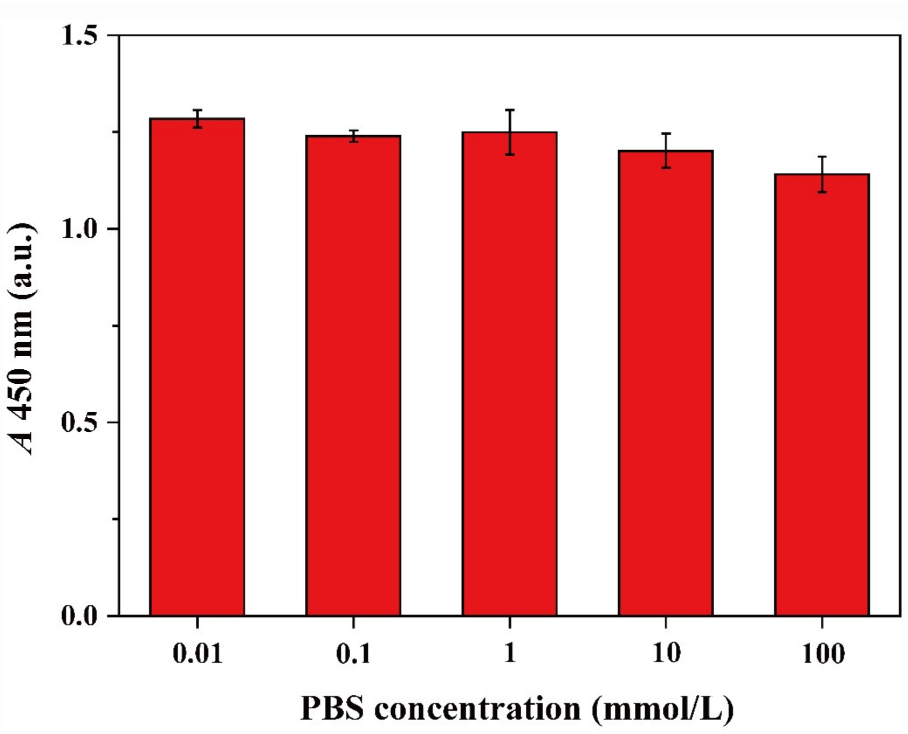

Fig. S4 Effect of salt concentration of buffer solution on the performance of the proposed method. Red bars indicated absorbance intensity (450 nm) (from left to right) for 2.0×10^5 CFU/mL *S. aureus* in buffer solutions over a range of PBS concentrations (0.01 – 100 mmol/L). Error bars indicated the standard deviations

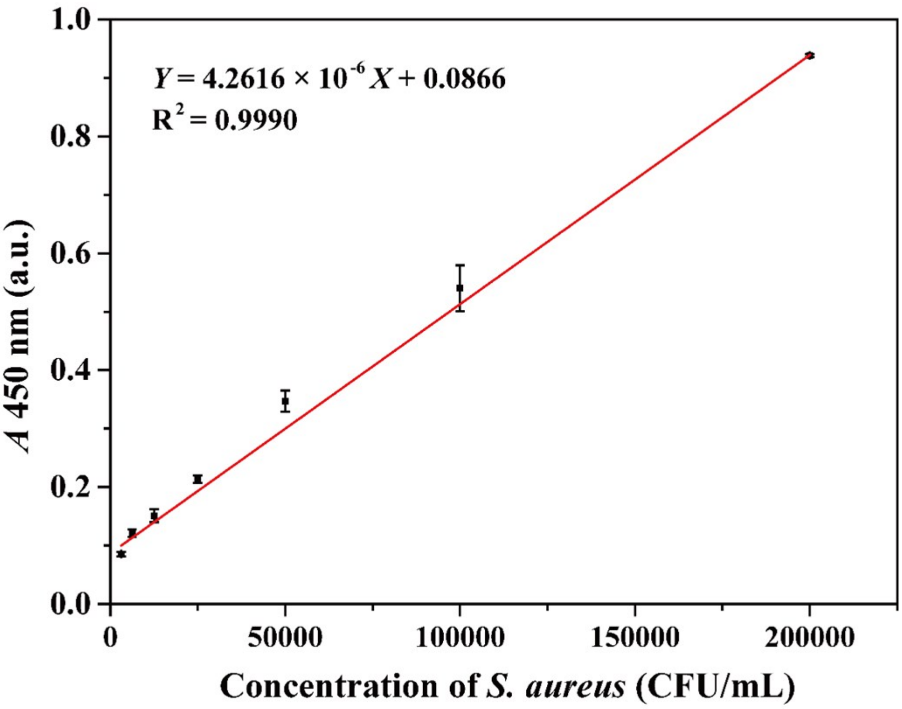

Fig. S5 Calibration curve of absorbance intensity (450 nm) along with *S. aureus* concentration under the optimal conditions in orange juice (the sample was 5-time diluted). Three independent measurements were taken from three individual preparations for each condition. Error bars indicated the standard deviations

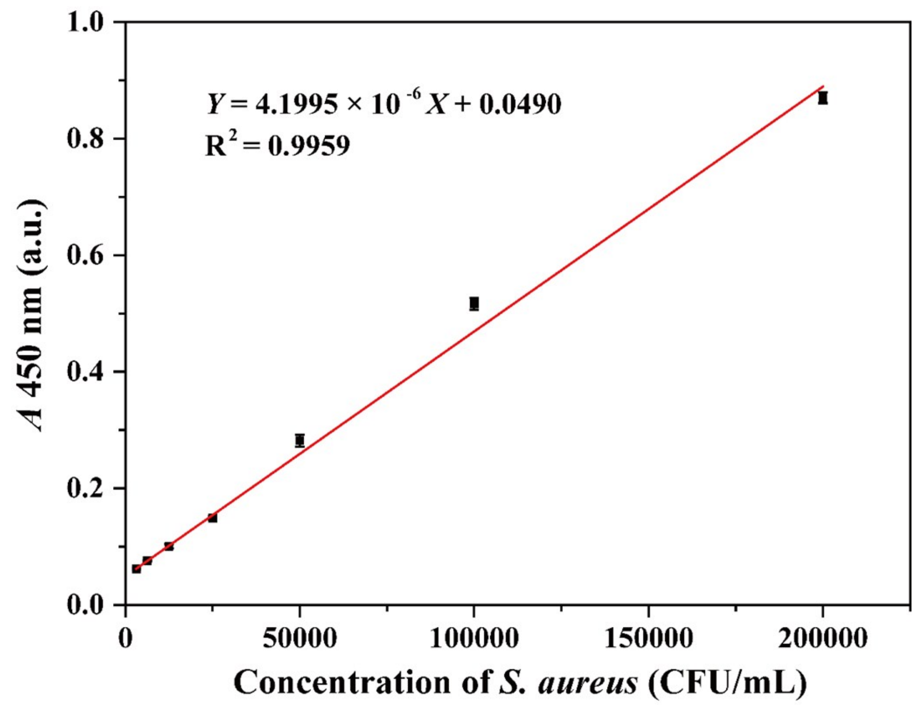

Fig. S6 Calibration curve of absorbance intensity (450 nm) along with *S. aureus* concentration under the optimal conditions in spring water. Three independent measurements were taken from three individual preparations for each condition. Error bars indicated the standard deviations

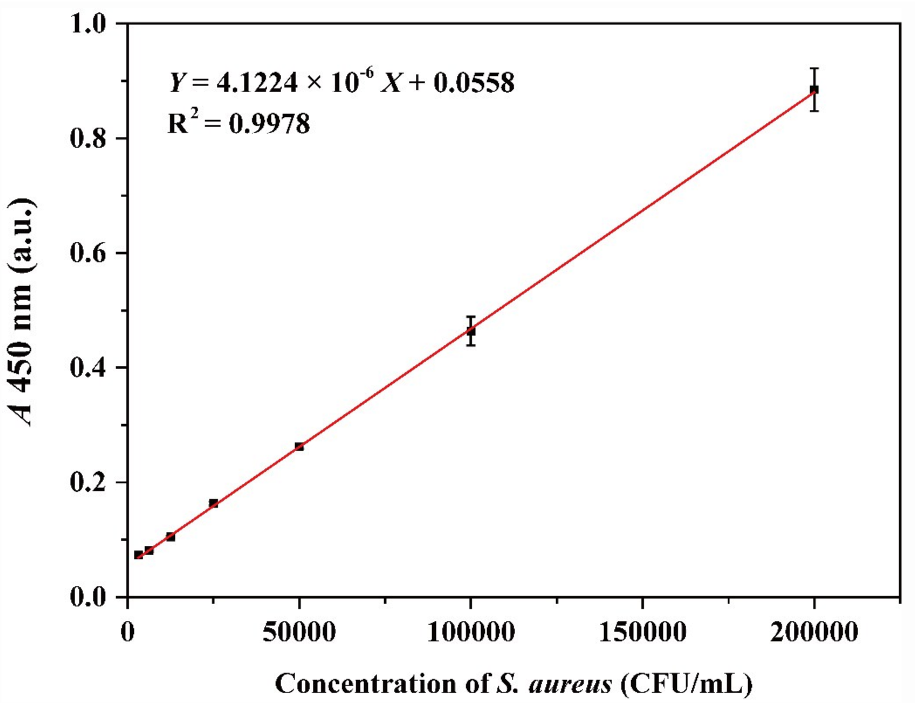

Fig. S7 Calibration curve of absorbance intensity (450 nm) along with *S. aureus* concentration under the optimal conditions in human urine. Three independent measurements were taken from three individual preparations for each condition. Error bars indicated the standard deviations

Table S3 Analytical results of *S. aureus* in real samples obtained using the proposed method and by ELISA (n=3)

Samples	Spiked ($CFU\ mL^{-1}$)	Found ($CFU\ mL^{-1}$)	RSD (%)	ELISA ($CFU\ mL^{-1}$)	RSD (%)
Orange juice [a]	1.3×10^4	1.4×10^4	6.32	1.2×10^4	2.31
	5.0×10^4	5.5×10^4	7.21	5.4×10^4	4.42
Spring water	1.3×10^4	1.2×10^4	6.87	1.1×10^4	2.21
	5.0×10^4	5.2×10^4	4.35	4.7×10^4	2.87
Human urine [b]	1.3×10^4	1.2×10^4	3.52	1.3×10^4	2.39
	5.0×10^4	5.0×10^4	1.75	5.5×10^4	1.09

[a] The sample was 5 times diluted. [b] The sample was 10 times diluted.

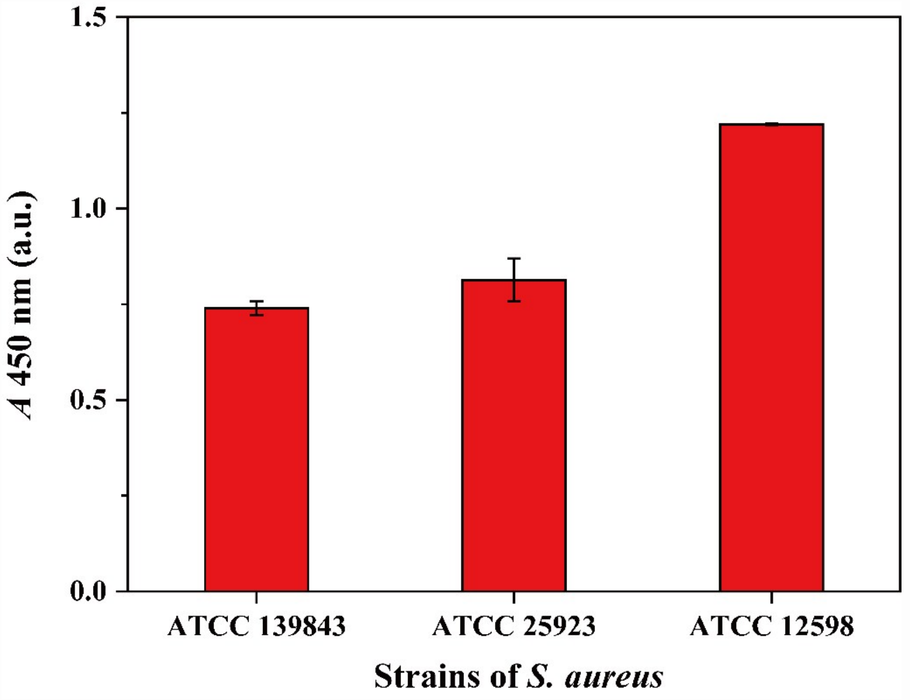

Fig. S8 Different strains of *S. aureus* detected by the proposed method. Red bars indicated absorbance intensity (450 nm) (from left to right) for 2.0 × 10^5 CFU/mL *S. aureus* (ATCC 139843), *S. aureus* (ATCC 25923) and *S. aureus* (ATCC 12598). Error bars indicated the standard deviations
